# Evidence for the Role of Magnetic Source Imaging in the Presurgical Evaluation of Refractory Epilepsy Patients

**DOI:** 10.3389/fneur.2019.00933

**Published:** 2019-09-10

**Authors:** Evelien Carrette, Hermann Stefan

**Affiliations:** ^1^Reference Centre for Refractory Epilepsy, Ghent University Hospital, Ghent, Belgium; ^2^Department of Neurology-Biomagnetism, University Hospital Erlangen, Erlangen, Germany

**Keywords:** magnetic source imaging, refractory epilepsy, presurgical evaluation, equivalent current dipole modeling, magnetoencephalagraphy (MEG)

## Abstract

Magnetoencephalography (MEG) in the field of epilepsy has multiple advantages; just like electroencephalography (EEG), MEG is able to measure the epilepsy specific information (i.e., the brain activity reflecting seizures and/or interictal epileptiform discharges) directly, non-invasively and with a very high temporal resolution (millisecond-range). In addition MEG has a unique sensitivity for tangential sources, resulting in a full picture of the brain activity when combined with EEG. It accurately allows to perform source imaging of focal epileptic activity and functional cortex and shows a specific high sensitivity for a source in the neocortex. In this paper the current evidence and practice for using magnetic source imaging of focal interictal and ictal epileptic activity during the presurgical evaluation of drug resistant patients is being reviewed.

Since the first MEG recordings in 1968 performed by Dr. Cohen using a single channel, the MEG technique has been optimized. The increase in the number of channels toward the whole head dewars with more than 300 sensors we use today, resulted in a breakthrough of MEG in the presurgical evaluation of patients with drug resistant epilepsy.

Using MEG in the work-up of epilepsy patients holds many advantages which are clear and multiple; just like EEG, MEG is able to measure the brain activity, and therefore the epilepsy specific information, directly (independent of blood flow), non-invasively and with a very high temporal resolution in the order of milliseconds. Thanks to its unique sensitivity to tangential sources it gives the full picture when combined with EEG, it allows accurate source imaging and shows a specific sensitivity for neocortical sources.

Typically patients with epilepsy who undergo MEG are in supine position during the recording session lasting in European centers about 90 min (range 60–420 min) and are encouraged to fall asleep or are even sleep deprivated (1).

In the MEG data recorded different features are being used to study the disease and more specifically to localize the epileptogenic zone (EZ) as precise as possible to plan surgical procedures in drug resistant epilepsy patients. Like stated in the position statement paper by the American Clinical MEG Society (AMEGS) MEG should be used as a non-redundant method to localize the EZ in people with drug resistant localization related epilepsy, especially if those cases were the standard and established presurgical evaluation modalities fail in providing sufficient information (2).

## Equivalent Current Dipole Modeling—Practical Guidelines

In this review mainly source localization obtained by equivalent current dipoles (ECD) is being discussed. This inverse solution localizes a point source assuming that all recorded magnetic signal is explained by a single dipole source. To check the reliability of this dipole several indicators are calculated for example the goodness of fit or the correlation coefficient.

ECD modeling is widely used for clinical source localization of interictal epileptiform discharges and today the only solution approved by clinical guidelines (2).

In contrast, when distributed methods are being used to perform magnetic source imaging, maps of the location and the extent of the generators are being displayed, however the yield of this inverse method has not been clinically validated yet and is therefore beyond the scope of this paper.

**Figure 1:**
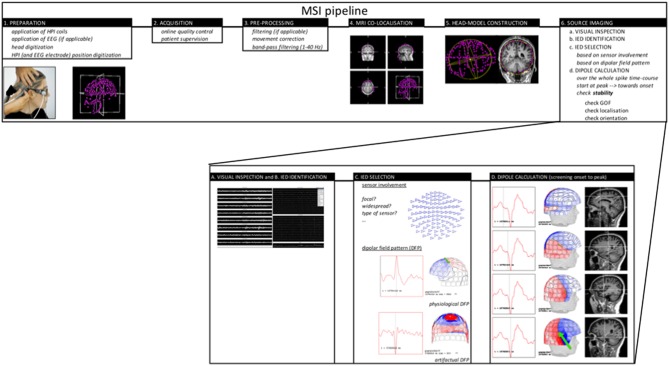
MSIAQ pipeline.

Today the proposed and accepted MSI pipeline to perform ECD modeling is illustrated in Figure 1. Based on the guideline provided by the American MEG Society (2) some elaboration on the following important steps in the pipeline should be mentioned:

- Visual identification of well-defined IED's is of major importance and this can include spikes and sharp waves. Research on the value of modeling slow-wave and/or fast activity is ongoing.
- The importance and difficulty of selecting a specific or several time-points in the IED waveform for source analysis. Typically, the peak of a spike-wave is being chosen as this time-point can guarantee a good signal-to-noise ratio (SNR) however might not represent the origin of the spike. Alternatively, a point on the rising phase of the IED should be checked and if SNR allows also the onset of the discharge. As described in the guideline, it is possible to trust the modeled spike-peak if the dipolar field pattern is stable (no rotation) over the whole spike-course. In case rotation of the field is evident, it is being suggested to look for an earlier source throughout the whole time-course of the spike to check for propagation. As SNR will be lower in this case, results need to be interpreted with more caution. Averaging might of course increase SNR (see lower).
- Evaluation of reliability of the ECD using solution parameters like goodness of fit, total error, coefficient of correlation or confidence interval are used however cannot guarantee appropriateness of the model. In case of ECD it is important to understand (1) the pro's and con's of the dipole model, (2) the character of the cortical spike sources, and (3) the current recommendations on MSI.
- Averaging of IED is not common or standard practice for MSI. It holds the advantage that SNR increases and therefore allows to model earlier phases of the IED time-course which might hold benefits (3), however it might blur differences in location or time course if similar IED are taken together nevertheless they arise from different and separated sources.

## The value of Interictal MSI

### Spike Yield and Sensitivity

Given the acquisition circumstances, the most common feature measured with MEG is the interictal epileptiform discharge (IED) rather than a seizure. Depending on the localization of the so-called “irritative zone” sensitivity to detect IEDs with MEG differ. The average reported sensitivity to detect clinically significant IEDs is about 75% (3–5).

Studies on simultaneously recorded scalp EEG and MEG comparing IED's, show a complementarity between both techniques. This complementarity is a result of the difference in sensitivity of EEG and MEG for radial and tangential sources in the brain. This difference is caused by the different orientation of the apical dendrites of the pyramidal cells in the gyri or sulci. MEG is selectively sensitive for sources that are tangentially orientated corresponding to the neurons on the banks of the sulci, whereas EEG on the otherhand is mainly sensitive for radially orientated sources corresponding to the neurons on the top of the gyri (and in a lesser extend also for tangential sources). This explains why both techniques, EEG and MEG should be considered complementary (6). Illustratively Ebersole and Ebersole state in their paper “that the brain without sulci, but with major fissures, is a simplified but reasonable, model of the cortical generator for scalp EEG and the brain “seen” by MEG appears to have no gyral crowns over the convexity but rather erode sulci and fissures” (7). Therefore, it is clear that the full image can only be “seen” by combining both techniques.

In literature investigating the sensitivity of EEG and MEG, it is being described that in more than half of patients IEDs can be identified both on scalp EEG and MEG, in 7% of patients only EEG show IED and in 18% only MEG show IED. In 21% of patients no IEDs can be recorded with any of the two modalities. Interestingly they additionally showed that 47% of patients who did not show IEDs on scalp EEG did had spikes during 1 h of MEG recording, supporting the performance of MEG in EEG negative patients (8–11). Duez et al. compered the number of epileptiform discharge (ED) clusters between MEG and (high-density) EEG (64–80 electrodes) and found that 72% of ED clusters were visible both in MEG and EEG, in 15% only on EEG and in 13% only in MEG. More than 1/4 of ED clusters was visible in only 1 modality showing the importance of simultaneous EEG and MEG recording (12). In the recent study by Plummer et al. comparing simultaneous recorded MEG and HD-EEG report IED only in HD-EEG in 42%, only in MEG in 16% and seen in both modalities in 42%. This somewhat different result compared to earlier studies (high number of IED reported only for HD-EEG) might be a result of the additional 12 electrodes that were placed inferior temporal which is not standard practice (13).

In addition, Ebersole and Wagner recently reported on the importance of taking into account “the number of spike “types” recorded by EEG and MEG in addition to the spike frequency.” They explain that this can only be done by combing EEG and MEG both for the recording and for the source modeling. They conclude that the absolute number of spikes can have some clinical significance, but that in the context of epilepsy surgery it is more important to identify the number of foci the spikes arise from. They showed that using only MEG would have let to missing at least 1 spike “type,” clear and evident in EEG, in almost 50% of the patients (14).

Studies have compared subdural recordings with simultaneous MEG recordings and showed that all MEG spikes had subdural counterparts, whereas 56% of the subdural recorded spikes were shown on MEG. However for lateral neocortical, insular, intra-sylvian, and (frontal) interhemispheric foci this percentage rose to 75–90% of spikes (5, 9, 15, 16).

With similar studies it was shown that in neocortical epilepsy MEG picks up IED that extend no more than 3–4 cm^2^ of activated lateral frontal neocortex on the subdural electrodes, up to 6 cm^2^ for more basal frontal and temporal neocortex whereas other studies showed that scalp EEG only detects IED when >10 cm^2^ of the neocortex is activated (17, 18). However, these studies did not use the HD-EEG set-ups available today.

Ideal is the combination of EEG and MEG to increase spike-yield (19). Indeed Heers et al. compared the spike yield in EEG, MEG and EEG/MEG following sleep deprivation and reported, respectively, 51, 60, and 71% IED detection (10).

Due to this high sensitivity for the cortical convexity, MEG has recently been claimed complementary with SEEG and subdural invasive EEG recording. Vadera and colleagues performed simultaneous MEG and SEEG. They showed that MEG was able to fill the gaps in-between the recorded brain activity from the depth electrodes and allowed a more tailored resection of only a small amount of brain tissue (20).

### Diagnostic Accuracy and Added Value

#### Patient Type

In epilepsy surgery the best outcomes are described for patients with mesial temporal lobe epilepsy (Engel I in up to 90%). This is not the case for patients with neocortical epilepsy. In these patients the current presurgical evaluation is not sufficient. The inclusion of MEG might be an important step as literature agrees on the fact that MEG is more sensitive for *neocortical sources* compared to deep sources.

Different studies have focused on the difficulties known in frontal lobe epilepsy (FLE) where scalp EEG is often not able to detect interictal or ictal activity due to fast propagation, muscle activity and source orientation whereas MEG could (21). They found that the resection of monofocal clusters in FLE and the tailoring of the resection by including clusters adjacent to the lesion correlated both significantly with good postsurgical outcome (22). Wu et al. retrospectively evaluated the correlation between semiology and MEG in seven patients with FLE and found in FLE that MEG non-invasively complemented the localization hypotheses obtained by ictal semiology (23). Ossenblok et al. optimized the procedures for localizing IED in FLE. Their conclusion was that MEG can be used as a “fast screening method for identifying the distinct categories of spikes and brain areas responsible for these spikes.” Moreover, the simultaneous recording of EEG and MEG allowed them to compare both modalities directly for FLE and showed superiority of the MEG spike yield and localization over EEG (16).

The *insular cortex* is a second region that often causes difficulties in the current conventional presurgical work-up. Mohamed et al. retrospectively looked into their 14 insular cases and compared the MEG, FDG-PET and ictal SPECT result to the resection margin. They described three different patterns of MEG spike sources, (1) posterior operculo-insular cluster, (2) anterior operculo-insular cluster or (3) no cluster but rather diffuse perisylvian distribution. In the patient group that underwent surgery and had an anterior operculo-insular cluster, MEG provided superior information to ictal SPECT in 4/6 patients and to interictal PET in 5/6 patients (24). Park and colleagues described an interesting case with insular epilepsy in whom the IEDs simultaneously recorded on EEG and MEG were best explained by ECD in the anterior temporal lobe as seen in patients with TLE. However, the IEDs that were only measured by MEG and not seen simultaneously on EEG were best explained by ECDs adjacent to the insular lesion. This case report shows the potential of MEG to detect insular activity that is undetectable by scalp EEG (25). Different studies confirmed the role of MEG in the identification of the epileptogenic zone (EZ) in insular cases by confirming results with intracranial monitoring or following resective surgery (26, 27). In addition Yin et al. report on the importance of non-invasively recorded HFO (rippels associated with spikes) with MEG which show to be valuable for the localization of the EZ in insular epilepsy. They showed that resection of insular tissue generating ripples during IED's was more successful then when tissue was resected that only generated IED, however this difference was not statistical significant (28).

Comparable reports are available for sources in the *fronto-parietal operculum* (29) and *medial occipital region* (30).

Nevertheless, MEG seems more sensitive for neocortical sources, studies did confirm that *mesial temporal* spikes can be detected by MEG and in these patients too MEG can add crucial information to the presurgical work-up. Kaiboriboon evaluated the ability of MEG to detect mesial temporal spikes and found a sensitivity of 86%. In 60% of patients with non-localizing ictal video-EEG monitoring (VEM) and 67% of patients with non-localizing MRI, MEG showed well-localized IEDs ipsi-lateral to the side of surgery (31).

*Non-lesional neocortical* patients form the most difficult group of patients for the planning of resective surgery. Only 35% of the non-lesional extra-temporal lobe cases are rendered seizure free following epilepsy-surgery (32). MEG has shown to be useful as a guide to identify very subtle lesions with and without post-processing techniques (see infra) or high-resolution (surface coil) imaging (33–35) or to implant patients with intracranial electrodes (36). Definitely when focal MEG clusters are observed, this is very valuable in the presurgical decision-making and shown to be a positive predictive factors for successful resective surgery (27, 36, 37). In this complex patient group, Jeong et al. compared MEG with other presurgical investigations and compared all to the intracranial golden standard. It was shown that in 86% of patients MEG lateralizes correctly. For ictal VEM this is the case for 78%, for PET 70%, and 57% for ictal SPECT. On a lobar level, MEG and ictal VEM correctly identified the involved lobe in 65% of cases, PET in 57% of cases and ictal SPECT in 52% of cases (38). In the study by Itabashi including patients with very subtle (initially missed) focal cortical dysplasia (FCD), it was suggested that “MEG-guided a posteriori review of MRI” should become a routine part of a clinical practice and definitely in the preparation for a multidisciplinary presurgical meeting. In this role MEG could contribute to avoiding invasive evaluations and lead to improved surgical outcome (33, 39). Aydin et al. suggest in their paper to combine EEG/MEG source analysis with high resolution zoomed MR imaging, limited to small areas centered at the EMEGS source location as a new diagnosis strategy (35).

Besides the important role of MEG in non-lesional cases, it also has an important value in *lesional cases*. Kim et al. showed that the number of MEG dipole clusters and the proportion of dipoles in the resection cavity was not associated with seizure free outcome for the whole group of children however for cases with localized neocortical MRI lesions MEG source localization successfully localized the peri-lesional epileptogenic zone (40). A few important epileptogenic lesions are Focal Cortical Dysplasias (FCD), cavernoma's and tubers in Tuberous Sclerosis. These will be discussed in more detail below.

A FCD is a highly and intrinsically epileptogenic lesion. Over 76% of patients with these lesions become intractable to AED however studies have shown that 50–70% of patients can be rendered seizure free following epilepsy surgery. Presurgical evaluation of these patients is therefore mandatory and MRI is as always important as it identifies these lesions by showing blurring of the gray-white matter, cortical thickening, and abnormal signs in the white matter (41). However, these abnormalities might also be microscopic and not visible or only subtle on optimal imaging. Many studies focus on the role of MEG in the identification of these subtle but highly epileptogenic lesions (27, 39, 42). Due to the intrinsic epileptogenicity of the lesion neurophysiology, and also MEG, plays an important role in the delineation of the extent of a FCD in the cortex (often beyond what is visible on MRI) and to predict the outcome following the removal of FCD lesions (41, 43, 44). Therefore the estimation of the spiking volume might be important like shown by Bouet et al. and classical equivalent current dipole models might fail to provide this estimation (45). FCD often generate (spike-independent) discharges in the beta-frequency-band. Heers et al. localized these discharges using Dynamic Imaging of Coherent Sources and found coherence between simultaneous MEG and intracranial EEG. The sources of the beta band activity localized within <2 cm of the epileptogenic FCD (46). In patients with FCD and MEG dipole clusters, the complete removal of the clusters is associated with good postsurgical outcome (38). Wilenius et al. described that in patients with MEG dipole clusters and Engel class I or II 49% of the clusters on average was removed, whereas the corresponding value in patients with Engel class III or IV was only 5.5% (42). Especially for FCD type II related epilepsy MEG showed to be a very strong tool. In the study by Kasper et al. MEG was combined with MRI post-processing techniques like for example MRI acquisition and morphometric analysis (MAP) and showed excellent surgical outcomes with 81% reaching Engel I compared to published series. The MEG sensitivity in this cohort was 95% in FCDII, compared to 70% reported from unselected epilepsy series (47).

Besides the associated refractory epilepsy, the high risk for bleeding makes cavernoma a clear indication for epilepsy surgery. Epilepsy is caused by the associated mass effect, gliosis and hemosiderine and therefore, in contrast to FCD, the tissue adjacent to the cavernoma, rather than the lesion, exhibits hyperexcitability. Studies in patients with cavernoma have shown that it might be important to perform more than a pure lesionectomy and for the delineation of the extent of resection needed, MEG might play an important role as you are able to map the epileptic activity on the structural image (48, 49). In case of multiple cavernoma MEG will mainly reveal the complexity but will contribute to the decision-making whether or not further invasive work-up is useful (48). Unfortunately in 20–40% of patients with cavernoma, multiple of these lesions can be identified.

Besides for cavernoma and FCD, MEG might play an important role in the presurgical evaluation of patients with tuberous sclerosis and brain tumors or in patients who need a second or third surgery when earlier procedures failed to control epilepsy. In post-operative situations MEG is superior over EEG because the magnetic field is not distorted by the skull defects. El Tahry and colleagues focused on the value of MEG in this patient population and compared MEG with ictal SPECT. They showed that MEG alone was successful in these patients after failed resective surgery. Only ictal SPECT with an early injection (<20 s) also had a good localization value (50).

#### Localizing Accuracy

In 2008 Lau et al. performed a systematic review of the available literature based on the *DARE scientific quality criteria* and concluded that “there was insufficient evidence in the current literature to support the relationship between the use of MEG in surgical planning and seizure-free outcome after epilepsy surgery” (51). However, this review received a lot of methodological critics.

Today the number of these specific studies comparing MSI result with the resection and postoperative outcome has only increased and therefore the evidence for its value in the presurgical evaluation became only more established. Very recently Mouthaan et al. performed an extensive meta-analysis on “the diagnostic accuracy and quality of evidence of interictal high resolution electric and magnetic source imaging (ESI and MSI) to localize the epileptogenic regions in the presurgical epilepsy evaluation.” The quality appraisal was based in a modified QUADAS-2 framework. Based on database searches they found almost 2000 abstracts that they screened and kept about 100 abstracts to do a full text assessment of which they excluded 47 articles for various quality reasons (no full text available, no study, different aim/objective, different index test, not the outcome of interest, …). For the other 51 articles they performed a full data extraction and quality appraisal. Only from 11 studies enough data could be captured to draw the anticipated conclusions [eight on MSI (236 patients) and three on ESI (127 patients)] as only studies without zero values in the 2 × 2 contingency tables were included. The study quality was however generally assessed as “poor” and no study was free of bias (selection of operated patients only, interference of source localization with surgical plan/decision, …) however they could conclude that the diagnostic accuracy analysis reveals for MSI and ESI a good surgical outcome in, respectively, 130/236 patients (54%) and 86/127 patients (67%). They additionally showed that the number of patients with a good surgical outcome is higher in the concordant group (76%) than in the non-concordant group (28%). There overall conclusion was that “both source localization techniques have a relatively high sensitivity (82%) and low specificity (53%) for the identification of the EZ. The diagnostic accuracy of MSI and HR-ESI to localize the EZ is strongly affected by poor study quality and likely biased toward overestimation therefore the results need to be interpreted with caution and independent support from other diagnostic tools is required to proceed to surgery. Higher quality studies, allowing unbiased MSI and ESI evaluation, are needed to judge results in light of source estimate size and resection size” (52).

Over the last 20 years, many studies investigated the role of MEG within the presurgical evaluation and confronted the MEG results with the golden standard available i.e., seizure outcome following resection and/or invasive recording and a few are described below.

Stefan et al. performed a retrospective study including 455 cases and concluded that MEG identified the correct lobe in 89% of cases and added information in 33% and crucial information in 10% (3). Papanicolaou et al. evaluated 41 patients that underwent MEG, IVEM, and resection. The seizure outcome was correlated to the overlap with the resection cavity and it was shown that IVEM was correct in 54% of cases and MEG in 56%. When groups were analyzed separately it was shown that MEG might be less beneficial relative to IVEM in ETLE compared to TLE (53). Knowlton et al. showed “a positive predictive value of MSI for seizure localization of 82–90% depending on whether computed against ICEEG alone or in combination with surgical outcome” (54). Knowlton et al. showed that a highly localized MSI result was significantly associated with seizure–free outcome for the entire surgical population (55). Kim and colleagues showed that the number of MEG dipole clusters and the proportion of dipoles in the resection cavity was not associated with seizure free outcome for the whole group however for cases with localized MRI lesions MEG source localization successfully localized the perilesional epileptogenic zone (40). Based on the retrospective analysis of the value of MEG performed at Cleveland between 2009 and 2012, Vadera et al. found that when preoperative MEG studies were fused with postoperative MRI, for 30/65 patients the MEG cluster was located within the resection cavity, for 28/65 completely outside the cavity and for 7/65 partially within. When postoperative outcome was analyzed they found that 74% of patients was seizure free at 1 year follow-up and 60% at 2 year follow-up. Correlation with the MEG result showed significantly improved likelihood of seizure freedom with complete clusterectomy in patients with localization outside the temporal lobe (56). Englot et al. reported on 132 surgical cases with at least 1 year post-operative follow-up of whom 70% had Engel I outcome. In 78% of cases MEG revealed IED and this result was compared with the (sub)lobe of resection, ECoG result and/or MRI lesion. They concluded that a concordant and specific MEG result predicted seizure freedom with an OR of 5.11 (57). The recent study by Duez et al. concluded that analyzing their combined dataset of MEG and EEG yielded significantly higher OR than separate analysis of both datasets, emphasizing the clinical importance of recording MEG and EEG simultaneously (12).

Nevertheless the recent paper by Plummer et al. focusses on the comparison between MSI, HD-EEG source imaging (EEG recording with coverage of the inferior temporal region with 12 additional electrodes) and simultaneous MEG and HD-EEG SI. In this paper they did not use the ECD model but used averaged data and distributed source modeling (sLORETA) and concluded, in contrast with their hypothesis, that independent source MEG and HD-EEG source imaging is superior to combined modeling (13).

In conclusion; based on different studies the clinical utility of MEG is ranging from 20 to 100% sensitivity and (3, 4, 51, 58, 59) from <10 to 100% specificity (51, 58–60). The positive predictive value is reported to be as high as 90% when compared to intracranial findings and association with surgical outcome (54, 60–63).

#### Therapeutic Impact and Added Value

The cornerstone investigation in the presurgical evaluation is scalp video-EEG monitoring (VEM). Despite the cheap cost of EEG, VEM is a rather expensive investigation, as it requires long-term admission at the hospital. In addition, MEG is a fast and more patient-friendly screening tool. Paulini and colleagues compared MEG with VEM and found that when long-term VEM gives insufficient localizing information, a (short) MEG session does in about half of patients (5). In 2004 Pataraia et al. investigated the added value of MEG compared to interictal and ictal VEM and used the surgery to confirm the results. In over 30% of cases MEG and VEM provided equivalent results however in 40% of patients additional information was available. When EEG was non-localizing MEG contributed to the localization of the region that was subsequently resected in 59% and when EEG was only partially localizing, MEG contributed significantly in 73% (4).

Different studies evaluated how the inclusion of MEG in the decision-making process changed or would have changed the patient management (Table 1). Overall studies reach a consensus that adding MEG to the presurgical evaluation protocol will change the management of about 1/5 up to 2/3 patients (depending on the inclusion level) (55, 62, 64–66). Very recently Duez et al. evaluated the effect of simultaneous EEG and MEG source imaging and revealed changed management in 34% of patients and these changes were useful in 80% (12). The type of patients included in the different studies can explain this broad difference in added value. Like mentioned in the study by Mohamed et al. mixing lesional and non-lesional cases for example just like combining the straight forward and “difficult” cases in one study like in the study by De Tiege et al. results in an underestimation of the added benefit of MSI. In contrary the study by Duez et al. only included the more complex cases which might lead to an overestimation (12). Overall the value of MEG (and EEG) source imaging is clearly large in especially the non-lesional cases.

**Table 1:** This table summarizes the outcome of studies focussing on the added value/effect of MSI on the decision making and/or management of patients in the presurgical evaluation.

**Study**	**Inclusion level**	**N^°^ PT**	**Change**	**Relevance**	**Remark**
Sutherling et al. (62)	All consecutive surgical candidates with neocortical epilepsy^*^	69	23 (33%)	6/29 (20%) of patients who eventually underwent resective surgery	^*^All pt meeting the criteria for direct temporal lobectomy or lesionectomy are excluded
Knowlton et al. (55)	All patients planned for intracranial work-up	77	18 (23%) (extra electrode coverage)	7/18 (39%) (seizure onset on the extra electrodes)	
De Tiège et al. (64)	All consecutive surgical candidates^*^	70	15 (21%) (44% of eTL-cases)	9/11 (82%)	^*^Including the straight forward cases
Ito et al. (65)	pt studied for clinical diagnosis and preoperative evaluation^*^	73	17 (23%)		^*^Only pt with IED were included
Mohamed et al. (66)	Consecutive non-lesional surgical candidates^*^	31	21 (68%)	12? (could be an overestimation)	^*^All patients underwent MEG but this was not taken into account at the time of the decision making. Retrospectively the lack of this information was assessed
Duez et al. (12)	Consecutive included patients in whom electromagnetic^*^ source imaging was part of the decision-making process (i.e., MRI negative or discordant other results)^**^	85	29 34%)	16/20 (with available results) 80%	^*^Simultaneous EEG and MEG source imaging ^**^Potential overrepresentation of the more complex cases

The study by Mohamed et al. could, due to their inability to provide reliable MEG results in a timely matter, assess the presumed impact of MEG retrospectively and in this way provide more clear evidence of the impact of MEG. They saw via this unique set-up that in 68% the management would have been different if the MEG results would have been available in time. In the subgroup of patients who in the meanwhile underwent surgery the inclusion of MEG in the work-up would have modified the resection in ~20% of patients possibly preventing negative outcomes and in another subgroup the unavailability of MEG led to a set of unnecessary/complicated intracranial recordings, surgical failures, and reoperations (66).

#### Guidance of Invasive Video-EEG Monitoring

The most important role of MEG today is the optimal guidance of invasive video-EEG monitoring (IVEM). IVEM is an invasive and expensive procedure associated with medical risks. However, for many patients it is their ultimate chance to be considered an eligible epilepsy surgery candidate. The planning of the implantation scheme is crucial and MEG has shown to be an ideal non-invasive investigation to guide this implantation especially in non-lesional cases (12, 36, 55, 66). Knowlton showed this elegantly in his study mentioned above (Table 1), including all patients planned for intracranial implantation. In this group in 23% MEG resulted in extra electrode coverage and in 39% of these cases these extra electrode-contacts involved the seizure onset (55). Also in the study by Mohamed it became clear that MEG is very important to optimally plan an intracranial implantation. Not only to make sure to end up with clinically relevant coverage of the seizure onset zone but also to minimize procedural risks to patients by allowing direct surgery without intracranial implantation, by reducing the number of contacts or excluding patients with a diffuse or inoperable epileptic area. This important role of source imaging within the presurgical evaluation, namely the optimal planning of the location of the intracranial electrodes, was confirmed in the recent study by Duez et al. showing changes in the location of the electrodes in 16.5% and offering the ability to implant electrodes in an additional 7% of patients that would not have been investigated without. In this study the source imaging allowed to skip intracranial recordings in 9.4% of patients and direct them to surgery immediately and withheld 1% of patients from undergoing surgical procedures (12).

The potential of MEG to identify the “primary irritative zone” via time-course analysis of the whole spike when interictal activity is complex (for example due to deep source) might be crucial in the planning of the IVEM, namely by predicting the patterns of spikes on ECoG or SEEG (67–70). Agirre-Arrizubieta compared 12 consecutive patients who underwent MEG before their implantation with electrodes with a control group that underwent an IVEM without MEG and were matched for implantation type. The groups were however not comparable when considering the complexity of the cases, as the MEG group consisted of more complex patients (and therefore underwent MEG). However no differences in number of successful implantations could be found between both groups suggesting that MEG can contribute to identify the ideal implantation site when standard methods fail (71). Still, a randomized study would be the only way to proof this with more certainty, however the value of MEG in the work-up is already to established, making randomization unethical.

## The Value of Ictal MSI

Besides by IEDs, epilepsy is characterized by the occurrence of seizures and until today the seizure onset zone (SOZ) has always been considered the closest approximation of the EZ. During MEG acquisitions the recording of seizures is difficult because the sessions are generally rather short (mean of 90 min according to a recent European survey) (1) and movement can cause problems recording good signal quality. Moreover no consensus concerning the best way to process magnetic ictal data has been reached because of the low signal-to-noise ratio during ictal activity, the different ictal discharge presentations and the evolvement of these patterns over time (72). Nevertheless the value of magnetic seizure activity has been described by different authors using different ways to analyze the data.

Sometimes the recording of ictal activity is rather a coincidence but in some centers it is being planned or anticipated. In a recent retrospective study including 377 patients who underwent a standard 1 h MEG, ictal MEG by coincidence (or by using known triggers like sensory or music) was found in 11% of patients (72).

First ictal MEG studies were performed with only a limited number of channels (73) or with multichannel hemispherical MEG recordings in combination with foramen ovale electrodes (74). Further ictal MEG studies showed that signal-to-noise ratio (SNR) at seizure onset the may be to low for dipole analysis. Often the typical movement related artifacts will obscure the seizure onset, however occasionally it was demonstrated that the ictal source localization was superior to interictal MEG correlating very nicely to invasively recognized seizure onset (75, 76). Later in time continuous ictal head movement measurement allows movement correction artifacts (77). Here instead of dipole analysis short time Fourier transformation (STFT) rhythm analysis was performed. In 63% concordant lobar interictal and ictal source localizations existed, again it was shown that ictal source localization was closer than the interictal source when compared to the seizure onset zone defined by invasive recording. In addition ictal MEG provided clear source localizations even if interictal MEG spikes were bilateral or missing. If interictal spikes were recorded bilateral than ictal recordings showed unilateral seizure activity.

In the recent retrospective paper it was shown that the resection of areas containing a minimum-norm estimate of a narrow band at onset, rather than a single equivalent current dipole, was associated with sustained seizure freedom. They also showed that ictal MEG patterns were clear when this was not the case with EEG showing also here a complementarity (comparable to the interictal situation). Moreover in patients in whom intracranial data were available the SOZ identified by ictal MEG recording correlated with the lobe of onset as identified via intracranial data in 88% (72).

Another group introduced gradient magnetic-field topography (GMFT) for the analysis of ictal discharges in patients with neocortical epilepsy after finding a higher spatial resolution in this patient group compared to the standard equivalent current dipole method (78).

Badier et al. compared SEEG epileptogenicity index, source localization using dipoles, and linearly constrained minimum variance (LCMC) (a beamformer technique). They showed that source imaging methods performed on rhythmic patterns were able to localize the EZ as validated by SEEG, but that LCMV was superior to ECD when concordance was compared (79).

The interictal MEG has a high sensitivity (0.95) and moderate specificity (0.75), ictal MEG has high sensitivity (0.96), and specificity (0.9) in predicting SOZ localization (80).

Finally it was shown that based on ictal-MEG, it was possible to change the management of patients initially considered unsuitable for surgery or planned for intracranial monitoring into candidates directly suitable for surgery with good postsurgical outcomes in those who were operated (81). A survey of comparisons of localizing accuracy using interictal and ictal MEG source localizations is provided by Stefan and Rampp (82).

## Other Features Recorded With MEG and of Value in the Presurgical Evaluation

Because not all patients show seizures or even IED during an MEG acquisition, alternative “features” are more and more often being studied. Not only slow activity (83) but also fast activity (84–87) has recently gained attention as well as network-analysis. Based on the current results it has been shown that it is possible to non-invasively identify regional interictal epileptic networks and their pattern of connectivity with MEG (70).

## Conclusion

Based on the review of the available literature patients who definitely need to be referred for magnetic source imaging are patients in whom a frontal, intrasylvian or insular focus is suspected, because MEG might be superior than EEG in localizing the irritative zone. Normal scalp EEG should not prevent patients from being referred for MSI and on the other hand neither should clear lesions on MRI prevent patients from being referred as MSI might help in the delineation of the resection needed beyond what is visible on imaging. It is clear that patients planned for an invasive video-EEG monitoring might benefit from MSI as it has been clearly shown that the implantation scheme can be optimized using the MSI-result.

In addition, in case of a focal MEG results in patients with normal imaging, MRI results need to be re-evaluated for subtle lesions guided by the focal MEG result. In case of patients with high seizure frequency it might be interesting to try to perform an ictal MEG as this can result in additional and accurate localizing information.

Besides these advantages specific limitations should be considered: Metal implants might cause problems, however specific filtering software might enable the interpretation of the signals. On the other hand the lack of IED (or seizures) during the MEG recording causes an inconclusive MEG result in up to 25% of patients undergoing MEG. Network-analysis like for example spike independent resting-state analysis might solve this problem in the future.

Just like all results within the presurgical evaluation, MEG should always be combined with the results of the other investigations and all results need to be interpreted with caution before the team can decide upon a next step. Today no unique presurgical tool is available to guide surgery and/or intracranial implantation on its own.

## Author Contributions

EC has written the review. HS has critically reviewed the drafts and added missing data.

### Conflict of Interest Statement

EC has received refunding for travel and registration costs and HS has received honoraria and travel reimbursement for lectures.
